# Medical Treatment in Heart Failure with Reduced Ejection Fraction: A Proposed Algorithm Based on the Patient’s Electrolytes and Congestion Status

**DOI:** 10.3390/medsci11020038

**Published:** 2023-05-24

**Authors:** Ioannis Paraskevaidis, Andrew Xanthopoulos, Nikolaos Karamichalakis, Filippos Triposkiadis, Elias Tsougos

**Affiliations:** 1 6th Department of Cardiology, Hygeia Hospital, 15123 Athens, Greece; 2 Department of Cardiology, University Hospital of Larissa, 41110 Larissa, Greece

**Keywords:** heart failure, sodium, potassium, pharmaceutical agents, algorithm

## Abstract

In heart failure (HF) with reduced ejection fraction (HFrEF), four classes of drugs (β-blockers, angiotensin-converting enzyme inhibitors/angiotensin receptor neprilysin inhibitors, mineralocorticoid receptor antagonists, and the most recent Sodium–Glucose Co-Transporters 2 Inhibitors) have demonstrated positive results in randomized controlled trials (RCTs). Nevertheless, the latest RCTs are not proper for comparison since they were carried out at various times with dissimilar background therapies and the patients enrolled did not have the same characteristics. The difficulty of extrapolating from these trials and proposing a common framework appropriate for all cases is thus obvious. Despite the fact that these four agents are now the fundamental pillars of HFrEF treatment, the built-up algorithm of initiation and titration is a matter of debate. Electrolyte disturbances are common in HFrEF patients and can be attributed to several factors, such as the use of diuretics, renal impairment, and neurohormonal activation. We have identified several HFrEF phenotypes according to their sodium (Na^+^) and potassium (K^+^) status in a “real world” setting and suggest an algorithm on how to introduce the most appropriate drug and set up therapy based on the patients’ electrolytes and the existence of congestion.

## 1. Introduction

In heart failure (HF) with reduced ejection fraction (HFrEF), four classes of drugs, the so-called fantastic four, also known as β-blockers, angiotensin-converting enzyme inhibitors (ACEIs)/angiotensin receptor neprilysin inhibitors (ARNIs), mineralocorticoid receptor antagonists (MRAs), and the most recent Sodium–Glucose Co-Transporters 2 Inhibitors (SGLT2is), have demonstrated positive results in randomized controlled trials (RCTs) [1,2]. However, the latest RCTs are not appropriate for comparison: they were carried out at various times with dissimilar background treatments, and the patients participating did not have the same characteristics. The difficulty of extrapolating from these trials and proposing a common framework that is good for all patients is thus obvious [3]. The difficulty is augmented in clinical practice given the hesitancy of clinicians to follow the schematic guidelines’ recommendations for several causes, such as intolerance connected to reduced blood pressure, heart rate, abnormal kidney function, or electrolyte disturbances [4]. These four agents are now the fundamental pillars of HFrEF treatment, but the built-up algorithm of initiation and titration is a matter of conflict and discussion [2]. In the majority of RCTs, patients with severe chronic kidney disease (CKD) are not included. However, we have to take into account that the definition of CKD is based on the estimated glomerular filtration rate (eGFR), which is not an independent measurement since patients with HF present with a different state of congestion and thus body weight instability, making the calculation of this variable problematic [5,6]. In this thesis, we propose a scheme of integration, which is what practitioners are requested to do according to the patients’ electrolyte profiles in an HFrEF setting.

Electrolyte imbalances are frequent in HFrEF patients and can be attributed to the use of diuretics, renal impairment, neurohormonal activation, or all of them. Sodium (Na^+^) and potassium (K^+^) are the most frequently affected electrolytes, but chloride is also affected, and hyponatremia is frequent in acutely decompensated HF and related to adverse events [7]. Hyponatremia can be attributed to dilution, true depletion of Na^+^, or both [8]. Hyperkalemia is also common, mostly due to side effects of the treatments given for renin angiotensin-aldosterone system (RAAS) blockade and potassium-sparing diuretics, whereas hypokalemia is connected to exerting diuretic use [7]. 

**Figure 1:**
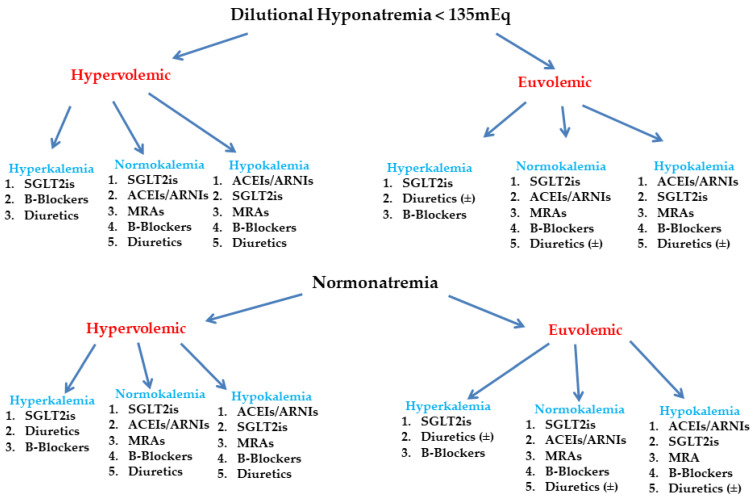
Algorithm for HFrEF treatment based on sodium (Na^+^) and potassium levels. Abbreviations: ACEIs—angiotensin-converting enzyme inhibitors; ARNIs—angiotensin receptor neprilysin inhibitors; MRAs—mineralocorticoid receptor antagonists; SGLT2is—Sodium–Glucose Co-Transporters 2 Inhibitors.

Our suggestion, depicted graphically in Figure 1, presents distinct patient profiles that should guide health care providers to personalize treatments according to HFrEF phenotype and drug characteristics. Each drug list depicts the priority of the drug to be titrated. Whether a HFrEF patient is congested or not can be assessed by medical history (i.e., dyspnea-orthopnea), clinical examination (i.e., hepatomegaly, jugular venous distension, edema), functional tests (i.e., a 6-min walking test), imaging (i.e., chest X-ray, echocardiography, lung ultrasound), and measurement of UNa in urine spot samples (i.e., values > 50–70 mEq/L denote effective diuresis and decongestion, whereas values <50–70 mEq/L require intensification of diuretic therapy) [1].

## 2. Common Sodium and Potassium Profiles in HFrEF Patients

### 2.1. Sodium Deflections in HFrEF Patients

Hyponatremia is a frequent electrolyte imbalance reported in hospitalized patients, with an estimated frequency of 15–28% [9,10]. It is defined as a serum Na^+^ ion concentration < 136 mmol/L. Mild to moderate hyponatremia is defined as serum Na^+^ levels 121–135 mmol/L, whereas severe to Na^+^ levels < 121 mmol/L [11,12].

Hyponatremia stems from a disproportion between body water and sodium. Based on the (patho)physiology, hyponatremia is categorized into dilutional and depletional forms. Dilutional hyponatremia, the most frequent type of this pathology, is caused by increased water retention while Na^+^ concentration is low, normal, or high [11]. Depletional hyponatremia results from a reduction in total body sodium due to renal, gastrointestinal, or blood loss [11]. Regarding the patient’s volume, dilutional hyponatremia can be either hypervolemic (i.e., HF) or euvolemic, whereas depletional hyponatremia is commonly hypovolemic.

Hyponatremia may be associated with a poor prognosis. The main cause of patients’ functional impairment is the occurrence of brain edema, which is associated with neurological symptoms, whereas mortality prevalence has been reported to be up to 50% of cases [13].

The exact burden of hyponatremia in HF is unknown [14]. The estimated prevalence is determined by several factors, such as the cut-off values used for its definition, the age of the patient, the functional classification of HF, and the periodicity of testing [15]. Notably, hyponatremia is generally underreported in hospitalized patients [16]. Based on the aforementioned limitations, it can be assumed that mild to moderate hyponatremia exists in 10% of those patients, whereas severe hyponatremia (serum Na^+^ levels between 128 and 130 mmol/L) is observed in less than 5% of cases [15,17]. Nevertheless, there are studies reporting higher levels of severe hyponatremia. For example, in a sub-analysis of OPTIME-CHF, approximately 1 out of 4 of the patients were in the lowest sodium quartile (i.e., serum Na^+^ concentrations 132–135 mEq/L), while in another sub-analysis of the ESCAPE trial, approximately 1 out of 5 of the patients had persistent hyponatremia (i.e., serum Na^+^ < 134 mEq/L) during their hospitalization [18,19].

The pathogenesis of hyponatremia in HF is multicausal. The impairment in cardiac output results in lower renal blood flow. Consequently, the decrease in glomerular filtration impairs the transport of solute and water to the distal diluting segment of the nephron. Concurrently, the heightened reabsorption in the proximal tubule intensifies the reduction of Na^+^ and water transport to the diluting sites. Therefore, renal capability to excrete dilute urine is flawed [15]. 

The reduction in cardiac output and in the effective circulating volume leads to activation of baroreceptors within the arterial tree, which trigger the sympathetic nervous system (SNS), the RAAS, and the release of arginine vasopressin (AVP). The heightened SNS activation increases Na^+^ and water retention [20]. Furthermore, angiotensin II leads to Na^+^ and water retention in two ways. Firstly, by raising the efferent arteriolar tone, thereby enhancing Na^+^ and water absorption by the final rise in the filtration fraction, and secondly, by stimulating the thirst center of the brain and therefore triggering the release of arginine vasopressin [15,21]. Hyponatremic patients with advanced HF often present with pathologically elevated plasma AVP levels that are resistant to acute water loading [22]. High AVP levels result in increased renal water retention by enhancing the number of aquaporin water channels in the kidney’s collecting duct after binding to the vasopressin-2 receptor subtype [20]. Lastly, HF diuretic therapy may also be a cause of hyponatremia [14,15]. Thiazide diuretics are a common cause, while non-thiazide agents, such as furosemide, spironolactone, and indapamide, have also been linked with hyponatremia [23].

Hyponatremia is a well-known prognostic indicator in HF [24]. Several studies have established the link between hyponatremia and adverse outcomes, both in patients with acute and chronic HF [25]. Hyponatremia is related to an increased rate of re-hospitalization, hospital resource use, complications, and increased costs [25]. However, since hyponatremia reflects pathophysiological mechanisms implicated in the HF syndrome (i.e., neurohormonal activation), a single improvement in serum Na^+^ concentration may alleviate patients’ signs and symptoms of HF but may not lead to better clinical outcomes [26].

### 2.2. Potassium Deflections in HFrEF Patients

Potassium (K^+^) deflections in HFrEF patients are frequent due to HF coexisting morbidities and HF-related treatments, but also because of HF itself as a syndrome of failing myocardium [27]. Both reduced and increased K^+^ can be potential lethal conditions by potentiating the risk of fatal arrhythmia, particularly in HFrEF patients [28]. 

HF patients frequently have multiple comorbidities and receive several medical treatments, further complicating K^+^ concentrations and management. The medical treatment of a HF patient includes neurohormonal inhibitors as well as loop and/or thiazide diuretics [1]. All of the above treatments may cause K^+^ deflections, leading to either hypokalemia or hyperkalemia. Comorbidities in HF patients such as aging, CKD, diabetes mellitus (DM), or frailty may result in serum K^+^ alterations, and these alterations can affect prognosis directly and also by restricting the use of life-saving medical therapies [27]. Hypokalemia is defined as serum K^+^ < 3.5 mmol/L and may be present in up to 1 of 2 patients with HF. Hypokalemia may result from the use of loop and thiazide diuretics. It may bring about fatal ventricular arrhythmias and elevate CV mortality. Its treatment encompasses the use of RAAS inhibitors, potassium-sparing diuretics, as well as oral K^+^ supplements [28]. Hyperkalemia is defined as serum K^+^ > 5 mmol/L and can be categorized as mild (>5.0 to <5.5 mmol/L), moderate (5.5 to 6.0 mmol/L), or severe (>6.0 mmol/L). It is linked to a high risk of morbidity and mortality. HF medical treatment (i.e., RAAS inhibitors) and comorbidities (i.e., CKD) may lead to hyperkalemia [23,27,28].

## 3. Pharmaceutical Agents in Heart Failure Patients and Their Effect in Sodium and Potassium

### 3.1. Sodium–Glucose Co-Transporters 2 Inhibitors (SGLT2i)

SGLT2i represents a novel class of drugs originally approved as antidiabetic agents but, after landmark trials, approved as HFrEF therapy [1]. The glucose co-transport protein is located in the renal proximal tubules, and SGLT2i promotes urinary glucose excretion by inhibiting glucose and Na^+^ reabsorption from there. However, the exact mechanisms of SGLT2i action in HFrEF remain a mystery, with several questions to be answered [29,30]. So far, a number of mechanisms have been suggested, including anti-inflammatory, antifibrotic, antioxidative, and antiapoptotic properties, with improved hemodynamics and ventricular unloading due to a reduction in blood pressure, weight loss, and arterial stiffness. SGLT2i has also demonstrated a reduction in uric acid levels and in epicardial adipose tissue with improved paracrine regulation of adipokines and decreased serum leptin levels [31]. They also improve cardiac energy as they boost glucagon utilization and shift metabolism from fatty-acid oxidation to glucose. Finally, the inhibition of sodium–hydrogen exchange via a direct cardiac effect can lead to a reduction in cardiac remodeling [29,32]. Regarding plasma Na^+^ and K^+^ levels, recent evidence suggests that treatment with SGLT2i does not prevent hyponatremia [33], whereas it reduces the risk of hyperkalemia but has a minimal effect on decreasing serum K^+^ [34].

SGLT2i are called “smart diuretics” as they lead to a sort of “pharmacological ultrafiltration” with a significant decrease in volume overload and enhancement of ventricular function, breaking the heart and kidney vicious circle. The effects of empagliflozin on clinical outcomes in patients with acute decompensated HF (EMPA-RESPONSE-AHF) trial was a randomized controlled trial that demonstrated that the use of SGLT2i empagliflozin (vs. placebo) increased urinary output until day 4 of hospitalization and reduced the combined endpoint of worsening HF, rehospitalization for HF, or death at 60 days [35]. Furthermore, the use of empagliflozin in the randomized EMPULSE (A Study to Test the Effect of Empagliflozin in Patients Who Are in Hospital for Acute Heart Failure) trial was associated with clinical benefit, defined as death from any cause, number of heart failure events, and time to first heart failure event, or a 5 point or greater difference in change from baseline in the Kansas City Cardiomyopathy Questionnaire Total Symptom Score at 90 days, as assessed using a win ratio, compared to placebo [36]. Therefore, similar to ARNI, there is enough documentation for an early initiation of SGLT2i in HFrEF patients and it should be used in combination with RAAS and β blockade. 

### 3.2. Beta Blockers

Beta-blockers are an established HFrEF therapy associated with improvement in symptoms, hospitalizations for HF, and morbidity [37]. Beta blockers and ACEIs can be administered simultaneously by the time the diagnosis of symptomatic HFrEF is confirmed, and no data supports commencing a beta-blocker prior to an ACEI and vice versa [1]. Beta-blockade should be started in clinically stable, euvolemic HFrEF patients at a low dose and steadily uptitrated to the maximum tolerated dose. In acutely decompensated HF patients, beta blockers should be initiated in the hospital with caution when the patient reaches hemodynamic stability. In patients suffering from atrial fibrillation (AF) and HFrEF, no benefit in outcomes has been demonstrated, but since these results originate from retrospective subgroup analysis and beta-blockers did not heighten any risk, administration applies to the AF patients also [1]. Beta-blockers prevent Na^+^ retention due to a blunting of the neurohormonal response [38]. In particular, a potential inhibition of renin secretion, which is in part beta-1 receptor-mediated, may affect the proximal reabsorption of Na^+^ via a decrease in angiotensin II production and/or an attenuation of the activity of the intra-renal sympathetic nervous system. An effect of beta-blockers on renin secretion may also modulate distal sodium reabsorption via aldosterone [38]. Hyperkalemia is an infrequent side effect of beta-blockers [39]. This might result from the inhibition of the sympathetic nervous system since β2 adrenergic agonists drive potassium into the cells by augmenting the activity of the Na^+^-K^+^ pump, and they also activate the inwardly directed Na^+^-K^+^-Cl^−^ cotransporter, a protein that enhances the active transport of Na^+^, K^+^, and chloride into cells [39].

### 3.3. Angiotensin-Converting Enzyme Inhibitors (ACEIs) and Angiotensin-Receptor Blockers (ARBs)

The use of angiotensin-converting enzyme inhibitors (ACEIs) in HF has been well established and is related to symptom improvement, prolonged survival, and reduced HF hospitalizations. ACEIs are recommended in all HF patients unless contraindicated or not tolerated [40,41].

ACEIs reduce the production of angiotensin II and degrade bradykinin. They are both mediators affecting the SNS, the vascular tone, the endothelium, and the myocardial performance. The hemodynamic effects of ACEI action include arterial and venous vasodilation, decreased systemic vascular resistance, a decrease in left ventricular filling pressure, and favorable ventricular remodeling [20,41].

Moreover, ACEIs have a selective efferent arteriolar vasodilatory effect, causing a mild to moderate reversible decline in renal function. They promote salt excretion and K^+^ retention by augmenting renal blood flow and reducing the production of aldosterone and antidiuretic hormone [20,41].

Angiotensin-receptor blockers (ARBs) are recommended for patients who cannot tolerate ACEI because of serious side effects [1]. Their effect on HFrEF has been variable, but unlike ACEIs, they have not demonstrated a reduction in mortality [42,43]. Serum electrolytes and renal function should be monitored during ACEI therapy, starting prior to therapy initiation, at 4 weeks, and after each significant increase in dose or clinical condition change [1]. ACEIs and ARBs increase the hazard of hyponatremia [44] and hyperkalemia (serum K^+^ > 5.5 mmol/L) [45].

### 3.4. Angiotensin Receptor/Neprilysin Inhibitors (ARNIs)

ARNIs are a novel class of drugs that altered the scene in HF treatment. They combine RAAS inhibition through angiotensin II receptor blockage by valsartan with neprilysin inhibition by sacubitril. Their action has proven to outperform enalapril in reducing mortality risk and hospitalizations in HF patients [46].

Neprilysin is a neutral endopeptidase involved in the degradation of endogenous vasoactive peptides such as natriuretic peptides, bradykinin, and adrenomedullin. Neprilysin inhibition gives rise to these peptides and reduces the neurohormonal overactivation that results in Na^+^ retention, vasoconstriction, and reverse cardiac remodeling [46].

To date, numerous clinical studies have demonstrated the safety and efficacy of sacubitril/valsartan, both in the context of chronic as well as acute HF. The PARADIGM-HF trial showed that sacubitril/valsartan was superior to enalapril in reducing hospital admissions for worsening HF, cardiovascular mortality, and all-cause mortality in patients with HFrEF with EF ≤ 40%. Additional benefits included an improvement in symptoms and QOL, a reduced incidence of diabetes requiring insulin treatment, reduced eGFR decline, and a lower rate of hyperkalemia [46]. Two studies, the PIONEER-HF and the TRANSITION trial, established the safety and efficacy profile of sacubitril/valsartan in decompensated hospitalized patients with reduced ejection fraction. Of note, some of these patients had not previously received ACEi or ARBs. Therefore, according to the 2021 ESC Heart Failure Guidelines, sacubitril/valsartan may be considered (class of recommendation IIb) in ACEi naive patients with HFrEF [1,47,48].

Early sacubitril/valsartan initiation in stable HFrEF patients, independently of ACEi/ARBs therapy, seems beneficial [49,50]. An adequate blood pressure (BP) and an eGFR ≥ 30 mL/min/1.73 m^2^ should be present in patients who are considered for sacubitril/valsartan initiation, and a washout period of at least 36 h in patients receiving ACEI treatment is mandatory to minimize the risk of angioedema [46]. ARNI may cause hyponatremia. Hyperkalemia is less common in patients treated with ARNI than those treated with ACEI/ARB, and ARNIs may attenuate the risk of hyperkalemia resulting from the intake of MRAs [51].

### 3.5. Mineralocorticoid Receptor Antagonists (MRAs)

Mineralocorticoid receptor antagonists (MRAs) are well established in the treatment algorithm for HFrEF patients. Their benefits in this group of patients were proven years ago in landmark studies for spironolactone and eplerenone, respectively, demonstrating a risk reduction in death and hospitalization [52,53].

These agents are administered to HFrEF patients regardless of their blood pressure, as their impact on blood pressure is minor. Furthermore, there are no limitations regarding the congestion status of the patients, and they can be introduced safely to the congestive patient before hospital discharge [4].

MRAs are also indicated in the subset of patients with increased arrhythmic burden, as they have been reported to decrease the risk of sudden cardiac death in RALES and EMPHASIS—although the reduction in EMPHASIS was not statistically significant [50,51]. Along with beta-blockers and ARNI, they compose a group of drugs with documented reductions in sudden death [54].

Caution is warranted when it comes to patients with HFrEF and hyperkalemia, as the use of MRAs can further increase serum K^+^ levels. Therefore, MRAs are administered carefully in patients with a serum K^+^ > 5.0 mmol/L. MRAs are currently contraindicated in patients with severe CKD (eGFR < 30 mL/min/1.73 m^2^ or creatinine level >2.5 mg/dL) due to a lack of data, as this was the exclusion limit for RCTs like RALES and EMPHASIS [50,51,53]. However, patients with eGFR between 30 and 60 mL/min/1.73 m^2^ are often not treated with MRAs, according to registries, due to fears of worsening renal function or hyperkalemia [55].

However, HF patients with mild to moderate CKD have been shown to benefit from MRAs. In fact, in a RALES sub-analysis that showed a 30% relative risk reduction for mortality, the patients who benefited the most were those with the lowest eGFR [52]. The EMPHASIS CKD-subgroup analysis also showed eplerenone to be “both efficacious and safe when carefully monitored, even in subgroups at high risk of developing hyperkalemia or worsening renal function” [55]. Based on this data, MRAs should be given to HF patients with non-severe CKD, provided that their renal function and K^+^ levels are closely monitored. Blood should be drawn at 1 and 4 weeks after initiating or increasing the MRA dose and on a frequent basis thereafter [4].

### 3.6. Potassium Binders

Neurohormonal inhibitors decrease adverse outcomes in patients with HFrEF but unfortunately increase the risk of hyperkalemia, especially for those with comorbidities such as CKD and/or DM [56,57]. Therefore, not surprisingly, physicians often prescribe suboptimal doses of the above-mentioned life-saving treatments to HFrEF patients due to the hazard of hyperkalemia. However, over the last few years, K^+^ binders have emerged as safe treatments for hyperkalemia in HF patients [58]. In particular, the administration of sodium zirconium cyclosilicate (ZS-9), a highly selective cation exchanger that entraps K^+^ in the intestinal tract in exchange for Na^+^ and hydrogen, as compared with placebo, led to a reduction in serum K^+^ levels in a cohort of ambulatory patients with baseline K^+^ levels of 5.0 to 6.5 mmol per liter [59]. The Hyperkalemia Randomized Intervention Multidose ZS-9 Maintenance (HARMONIZE) was a phase 3, randomized, double-blind, placebo-controlled trial evaluating ZS-9 in outpatients with hyperkalemia (serum K^+^ ≥ 5.1 mEq/L) [60]. The study showed that ZS-9 lowered serum K^+^ to normal levels within 48 h compared with placebo, whereas all 3 doses of ZS-9 resulted in lower K^+^ levels and a higher percentage of patients with normal K^+^ levels for up to 28 days [60].

Patiromer, a non-absorbed, sodium-free K^+^ binding polymer that exchanges calcium for K^+^ in the gastrointestinal tract, led to a reduction in serum K^+^ levels, as compared with placebo, as well as a decrease in the recurrence of hyperkalemia in a cohort of patients with CKD who were receiving RAAS inhibitors and who had serum K^+^ levels of 5.1 to less than 6.5 mmol per liter [61]. More recently, the randomized DIAMOND (Patiromer for the Management of Hyperkalemia in Participants Receiving RAASi Medications for the Treatment of Heart Failure) trial demonstrated that patiromer use was safe in 1642 patients with HFrEF and RAASi-related hyperkalemia, and it was related to significantly lower hyperkalemia episodes, concurrent use of high doses of MRAs, and overall higher RAASi use [62,63].

In conclusion, the administration of K^+^ binders may not only lower the risk of hyperkalemia, but it also assists in the treatment of hyperkalemia in high-risk patients such as those with HF who receive RAAS inhibitors (especially MRAs).

### 3.7. Diuretics

Current guidelines recommend the use of intravenous loop diuretics to ameliorate symptoms of fluid overload in patients with acutely decompensated HF [1]. Loop diuretics act by competing with chloride to bind to the Na-K-2Cl (NKCC2) cotransporter at the apical membrane of the thick ascending limb of the loop of Henle and blocking the cotransporter, which inhibits the reabsorption of Na^+^ and chloride. Therefore, loop diuretics increase renal Na^+^ and water output and consequently alleviate the symptoms of congestion [64]. However, the use of loop diuretics is associated with electrolyte disturbances such as hyponatremia, hypokalemia, hypochloremia, and hypomagnesemia [65]. Torsemide has been reported to lead to fewer electrolyte disturbances compared to furosemide; however, the recent TRANSFORM-HF randomized clinical trial revealed no significant difference in all-cause mortality among patients discharged after hospitalization for HF (torsemide vs. furosemide) over 12 months [66].

Despite the use of high-dose loop diuretics, many patients are discharged from the hospital with residual clinical signs of volume overload (i.e., diuretic resistance), a strong predictor of poor outcome [67]. The recent randomized Acetazolamide in Acute Decompensated Heart Failure with Volume Overload (ADVOR) trial showed that the use of acetazolamide (a carbonic anhydrase inhibitor that reduces proximal tubular sodium reabsorption) on top of furosemide was associated with a greater incidence of successful decongestion, defined as the absence of signs of volume overload, within 3 days after randomization and without an indication for escalation of decongestive therapy, compared to placebo, in hospitalized patients with acute decompensated HF [68]. However, patients who were on SGLT2i were excluded from the study.

Although sequential diuretic therapy has been suggested as a more effective decongestive strategy than loop diuretic therapy alone, current evidence regarding effective diuretic agents and administration schedules is limited [64].

## 4. Discussion

The latest ESC guidelines for HFrEF suggest initiation of ACEI, beta blockers, and MRA in all patients and uptitration to the doses used in the clinical trials [1]. ARNIs should be given to suitable symptomatic patients despite ACEi treatment. The new kids on the block, SGLT2i, should be added to therapy with ACE-I/ARNI/beta blocker/MRA unless contraindicated or not tolerated; dapagliflozin or empagliflozin are recommended for all patients with HFrEF regardless of the presence or absence of diabetes [1].

In RAAS blockade agents, titration should be performed in not less than 2 weeks, and electrolytes and renal function should be checked 1–2 weeks after dosage change. Beta blockers should be administered in stable HFrEF patients and carefully in NYHA IV patients or those recently exacerbated with HF [1].

SGLT2i should be administered to improve QOL, reduce the risk of HF hospitalization, and increase survival in HFrEF patients, regardless of concomitant DM. They don’t require dose titration, but as they may intensify diuresis, particularly when accompanied by Sacubitril/Valsartan and diuretic therapy, fluid balance must be monitored regularly [1]. In the convention set up, ACEI and beta blockers start together, followed by MRA, ARNI, and finally SGLT2i, with uptitration to target doses typically requiring 6 months or more. In another proposal by McMurray and Packer, HFrEF patients should be administered beta-blockers and SGLT2i as initial treatment, followed by ANRI and MRA, after cautious electrolyte and renal function monitoring. In this proposal, all steps are achieved, ideally within 4 weeks [3]. However, it must be considered that despite a plethora of guidelines and position papers, it remains a fact that many times it is challenging to follow these proposals.

In this respect, and in the era of both guideline and/or personalized medicine, it is worthy to combine these two strategies in order to give the medical treatment the opportunity to find the optimal path. Therefore, we propose an algorithm that aims to add additional value to the existing knowledge regarding HF treatment. Indeed, when we add the electrolyte deflections, which are common in HF patients, to our opinion, we strive for a more appropriate therapy.

In conclusion, it seems that profiling HFrEF patients and their respective therapies is of great value, and algorithm-based instructions are key for clinical practice.

## Data Availability

Not applicable.
